# A Conversation with Mona Minkara

**DOI:** 10.1021/acscentsci.3c01101

**Published:** 2023-09-27

**Authors:** Julie M. Nyman

At age 7, Mona Minkara was diagnosed with macular degeneration and cone–rod dystrophy, which eventually led to blindness. A doctor “point-blank told my mom that it wasn’t worth spending a penny on my education,” she recalled years later as she delivered a speech at her commencement from Wellesley College.

**Figure:**
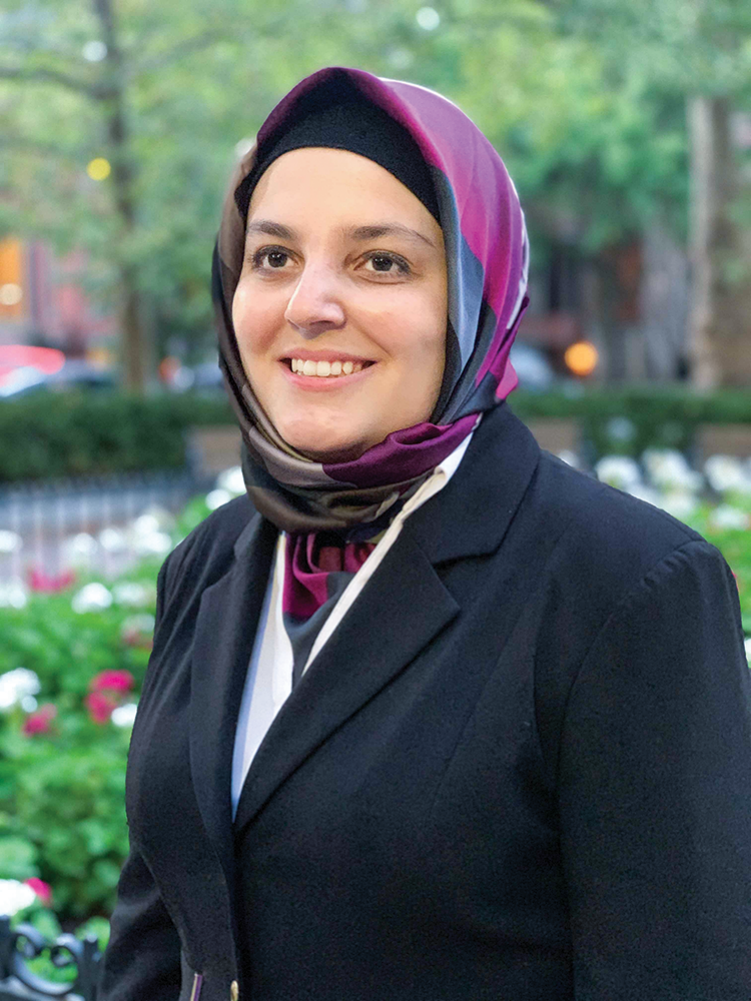
Credit: Courtesy of Mona Minkara. *Alt text: Gaze uplifted, Mona Minkara smiles. She is wearing a black blazer over a white dress shirt, and a delicately wrapped purple-and-gray hijab. Out of focus in her background is a vibrant green garden with white and pink flowers, framed by trees*.

Minkara completed her Ph.D. in chemistry at the University of Florida, and in 2019, she joined the faculty at Northeastern University in Boston. Her lab uses computational simulation techniques to investigate chemical interactions at the air–water interface in the lungs. The lab aims to simulate pulmonary surfactants—naturally occurring mixtures of proteins and lipids that coat the inside of the lungs’ winding passageways and keep them from collapsing. Drug developers could one day use the simulation data to possibly develop better therapeutics.

Minkara is using her platform as a professor to share tools that scientists can use to make the lab more accessible. In 2021, she was named the director of Science in Braille, a group of scientists who are blind and advocate for the inclusion and agency of blind people in science, technology, engineering, and mathematics (STEM).

Julie M. Nyman spoke to Minkara about her journey as a blind chemist and efforts to provide all scientists with the tools to succeed.

## What inspired you to do chemistry?

Ever since I was a child, I wanted to do science. So I watched *The Magic School Bus* as a kid and *Bill Nye the Science Guy*. And I thought, “This is amazing. This is what I want to do.” And then I was diagnosed, and everybody was like, “She can’t do it.” And I thought, “Why not?” So I just continued down that path, and I found myself in computational chemistry. And I loved it. And I took physical chemistry, and I was like, “This is it. I love quantum.”

## Did you ever feel like you had to prove yourself?

All. The. Time. Until a few years ago. I almost felt like I needed to overcome my blindness.

## When did you stop feeling that way?

I do not know if I fully stopped. I think it is a lot less now—it goes with age. Really what I needed to do is see the advantage of my blindness. I call it the unseen advantage. That vision is more than sight.

My postdoc adviser [J. Ilja Siepmann of the University of Minnesota Twin Cities] really helped. He specifically sought me out.

He believed that because I am blind and receive and process information differently than most people, I would be able to solve problems that others could not. And I did! By sonifying the data, I picked up on patterns my colleagues missed. We published a paper with my work (*Biochemistry* 2015, DOI: 10.1021/acs.biochem.5b00078).

I realized [in collaboration with the lab of Bryan Shaw at Baylor University] that making scientific visualizations into tactile tools can aid some scientists by creating multisensorial data that diversifies the ways the scientists can process information (*Sci. Adv.* 2022, DOI: 10.1126/sciadv.abq2640). Other tools that scientists can use to process data in nontraditional ways can be found on my web page Blind Scientist Tools.

**Figure:**
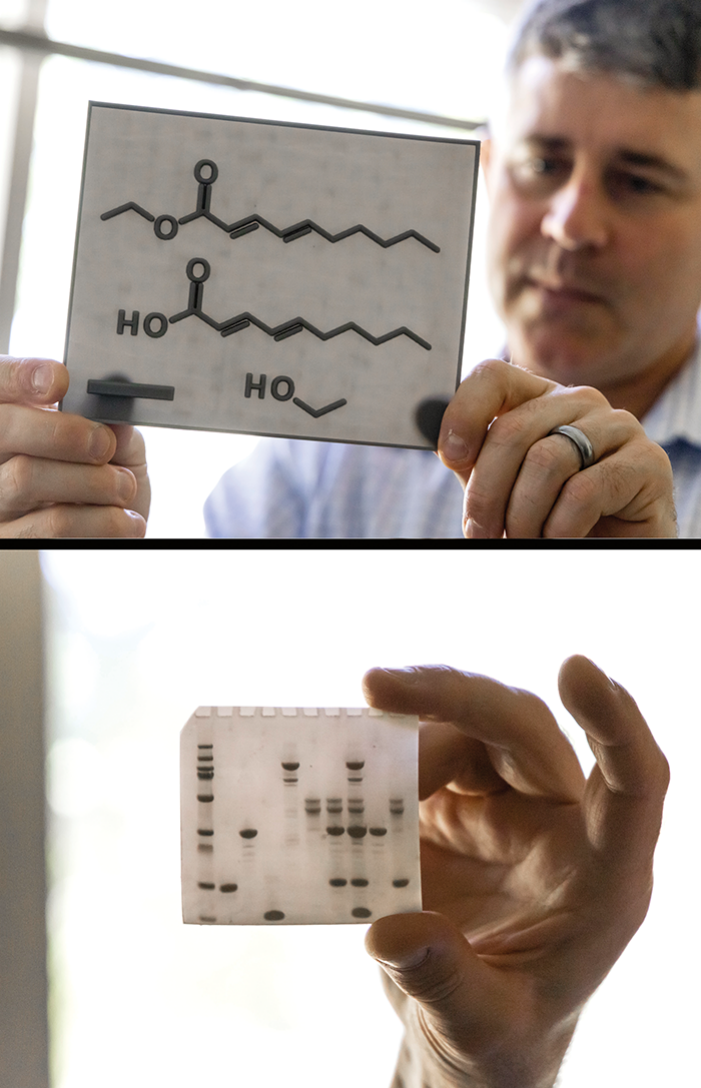
Bryan Shaw of Baylor University holds textured 3D-printed tactile graphics of a chemical structure (top) and an electrophoresis gel (bottom). Lifted ridges on the cards allow scientists and students to read information and data without having to see the graphics. Minkara collaborated with Shaw’s group on the work. Credit: Baylor Photography. *Alt text: Above, Bryan Shaw looks pensively upon a plastic 3D-printed card that he’s holding in his outstretched arm. It shows the chemical line structure for (2E,4E)-deca-2,4-dienoic acid and its ethyl ester as well as ethanol. The line structures are printed as raised ridges on a translucent plastic card. Below, he holds a similar plastic card that shows an image 10-lane electrophoresis gel with the bands represented by tiny raised blobs that appear darker on the card*.

## Can you tell me more about those tools?

I have a page on my web site called Blind Scientist Tools. Click on “Blind Scientist,” then click on “Tools,” and that highlights every tool I’ve ever used in different stages of my life.

In grad school, I really started compiling it. Now it is a massive web site, right?

## You have a lot of resources on there. It is very awesome. What resources were helpful to get you through school and university?

That is a complicated question: a lot of different tools. And as I went through my education path, I learned more and more of what could be helpful. But when I was in public school, the biggest tool I had was an aide in the classroom who read to me. And there really was not much else.

I did a lot of audiobooks back then. Then in undergrad, I started to face resistance. When I was a child, no one advocated for me to learn braille. Also, it was during a phase in which a lot of people were saying that braille was not as necessary as it used to be because audio can substitute, which was a big mistake. But I learned it as an adult. I read it now very slowly.

## What tools do you use most now? And what needs are still unmet?

Integrating the swell-form machine into my daily research routine has been a game changer. It helps me visualize basic diagrams. The swell-form machine is a heat lamp that reacts with a specific type of high-carbon ink, so after a piece of paper with this ink goes through the heat lamp, the ink swells and becomes tactile. It makes it extremely fast and easy to review graphics like simple graphs, charts, and maps.

I believe one of the biggest unmet accessibility needs in STEM is how inaccessible most PDF forms are to screen-reading software. I advocate for publishers to require authors to include alt text descriptions with their submissions so that they can be picked up by screen readers.

I really believe in sharing the kind of things I figured out so not everybody has to reinvent the wheel. I have a contact-me link, and people contact me from all over the world, especially blind individuals. These people are interested in STEM, and I meet with them on Friday afternoons. I meet with families, from kids to adults, and everything in between. People email me introducing themselves and ask for mentorship with their educational or professional journeys, and I often have mentorship meetings. I want more blind people to have the access to fulfill their dream to become a scientist.

## Do you primarily advocate for people who are blind, or do you eventually want to expand your advocacy to people with other disabilities?

I’ll advocate for anybody who wants to be advocated for, but I know the most about being blind. I was just on the planning committee for the National Academies of Science, Engineering, and Medicine and put out a conference called Disrupting Ableism and Advancing STEM. It was about advocacy for people with disabilities in STEM.

## I also wanted to ask you about your role as director of Science in Braille. How did that begin?

The executive director of the Royal Academy Science International Trust, Her Royal Highness Dr. Nisreen El-Hashemite, found me, and she was like, “I want to progress your mission, and I want to support making science accessible to the blind. Let’s create a global campaign.” So it is really she who brought it together.

In February of this year, the Science in Braille members spoke at the 8th International Day of Women and Girls in Science Assembly at the United Nations. There’s a great unmet need to connect students and professionals of STEM who are blind or partially sighted by forming communities that can help each other with resource sharing and advocacy.

## I saw that you got a standing ovation when you spoke at the UN. Clearly, you are thinking about dismantling the negativity around disability. What do you think is the best way for the global community to shift their perspective on blindness and disability?

The global community needs to realize that we are individuals and that they need us to contribute. Right now, global society sees us as burdens or people that need to be saved and rescued. Everybody has potential to contribute if we are given the right tools.

*Julie M. Nyman is a freelance contributor to*Chemical & Engineering News*, the independent news publication of the American Chemical Society. Center Stage interviews are edited for length and clarity.*

